# Legionnaires’ Disease on the Rise in Switzerland: A Denominator-Based Analysis of National Diagnostic Data, 2007–2016

**DOI:** 10.3390/ijerph17197343

**Published:** 2020-10-08

**Authors:** Fabienne B. Fischer, Claudia Schmutz, Valeria Gaia, Daniel Mäusezahl

**Affiliations:** 1 Swiss Tropical and Public Health Institute, 4001 Basel, Switzerland; f.fischer@swisstph.ch (F.B.F.); claudia.schmutz@swisstph.ch (C.S.); 2 Faculty of Science, University of Basel, 4002 Basel, Switzerland; 3 National Reference Center for Legionella, Service of Microbiology, Ente Ospedaliero Cantonale, 6500 Bellinzona, Switzerland; Valeria.Gaia@eoc.ch

**Keywords:** *Legionella* spp., disease surveillance, underestimation, Legionnaires’ disease, diagnostics, denominator data

## Abstract

The risk of falling ill with Legionnaires’ disease (LD) is suggested to increase, but the global burden of disease is unknown due to a lack of appropriate diagnosis and surveillance systems. In Switzerland, the number of LD cases, captured by the National Notification System for Infectious Diseases, has more than doubled since 2008. This study aims to investigate this increase, contextualizing disease surveillance data with denominator data, which is not routinely available, i.e., the number of tests performed for *Legionella* spp. We collected the testing data for *Legionella* spp. of 14 Swiss diagnostic laboratories and calculated the positivity, defined as the proportion of the number of positive tests to the number of tests performed. The number of positive tests increased proportionally to the number of tests performed; hence, the positivity remained stable. However, the cause of the increase in test volume is unclear and has a large impact on the interpretation of the positivity curve. Further, the test outcome was found to be dependent on regional determinants, and the diagnostic method applied. The lack of understanding if and at which stage LD is considered in current case management of pneumonia patients limits the interpretation of observed heterogeneities in incidence or underestimation of LD in Switzerland. The absence of (or non-adherence to) existing guidelines and the heterogeneity in diagnostic testing hampers the comparison of data in the Swiss public health context. Therefore, diagnostic procedures should be harmonised across Switzerland and adherence to national LD management guidelines supported.

## 1. Introduction

*Legionella* spp. are the cause of a group of diseases termed “legionellosis” ranging from mild and self-limiting Pontiac fever to potentially fatal Legionnaires’ disease (LD), characterized by pneumonia [1,2]. Infections with *Legionella* spp. occur through inhalation or aspiration of contaminated water or aerosols. In recent years, person-to-person transmission was also suspected [3]. Cases can occur sporadically, in clusters and large outbreaks.

Although *Legionella* spp. occur worldwide, the global burden of disease is unknown due to the lack of appropriate diagnosis and/or surveillance systems in many countries. In Europe in 2017, 1.8 cases per 100,000 population were estimated, corresponding to 9238 cases in total. In the same year, the US reported 7500 cases, corresponding to 2.3 cases per 100,000 population [4,5]. Case numbers have been increasing in European countries and the US in the past years.

In Switzerland, infections with *Legionella* spp. need to be reported to the National Notification System for Infectious Diseases (NNSID), which is managed by the Federal Office of Public Health (FOPH), since 1988. While all laboratory-confirmed infections are notifiable, only LD cases—cases with pneumonia—are considered as confirmed or probable cases, which are reflected in the numbers published in official statistics. The case numbers continuously increased from 219 in 2008 to 464 cases in 2017 [6].

The increase of LD cases in Switzerland, the rest of Europe and the US is not well understood. It has been hypothesised that the increase in incidence is due to augmented susceptibility in the population, climate change or changes in energy policies [4,6]. Common risk factors for LD are age > 40 years, being male, tobacco smoking, travelling abroad or having chronic conditions, e.g., diabetes mellitus or a compromised immune system [7,8]. Furthermore, several studies link weather and climate, namely warm and humid conditions, to LD incidence [9,10,11,12]. Efforts in energy saving, resulting in recommendations to lower temperature thresholds of potable warm water, could have the drawback to promote conditions which favour *Legionella* spp. proliferation [13].

Conversely, the increase in case numbers could also be an artefact. Increased awareness of physicians could lead to increased testing and hence, to more cases found. The incidence of legionellosis is generally thought to be underestimated; a study from Germany in 2008 estimated about 15,000 to 30,000 cases of sporadic LD annually [14]. Improvements in diagnosis and surveillance could lead to higher but more accurate case numbers [15,16].

We collected testing data of 14 Swiss diagnostic laboratories between 2007 and 2016 to evaluate the effect of changes in test numbers and diagnostic procedures on the notification numbers in Switzerland. Using this data, we calculated the positivity of *Legionella* spp. testing, emphasising on temporal trends, and assessed the determinants for a positive test outcome.

## 2. Methods

The methods of a positivity study have been described in detail elsewhere [17]. In brief, we collected testing data from 14 Swiss diagnostic laboratories. The laboratories were selected in 2016, based on providing most LD notifications in the prior 10 years.

We collected data on all tests performed for *Legionella* spp. regardless of the test outcome, between January 2007 and December 2016. Information requested included “date of test”, test result (binary; 0 = negative, 1 = positive), diagnostic test method, sample material used, patient identification number, and patients’ date of birth, sex and canton (a political and administrative subdivision of Switzerland, in total 26 cantons) of residence. The test result was reported by the laboratories and not assigned by the study team, hence the application and description of the case definition were not needed in this study.

We excluded tests of patients with residency outside of Switzerland, inconclusive test results, duplicated entries as well as “repeated tests”. Repeated tests were defined as more than one test performed per patient and disease episode. The definition of a disease episode was complex given the laboratory data available; the process is described in the Supplementary Material (see Supplementary File 1 and Figure S1 for details).

We use the term positivity as the proportion of the number of positive tests to the total number of tests performed for *Legionella* spp. [18,19]. The analysis was planned a priori and was conducted using STATA 15 (StataCorp., College Station, TX, USA). The positivity was calculated for different age and sex groups, test methods, sample materials, spatial (region and laboratory) and temporal (annual and seasonal) trends. The main outcome, the annual positivity, was age- and sex-adjusted using direct standardisation with the sample population (2007–2016) as the reference population.

We used mixed-effect logistic regression to account for clustered data to analyse the determinants for a positive test result. The significance level was defined as α = 5%. Univariable logistic regression was used to test the association between the test result and test year, season, time trend, sex, age group, laboratory, test method, sample material and greater region (Table 1). “Season” was modelled using sine and cosine functions with an annual period. The time trend was a continuous variable combining test month and test year. The age groups were based on categories (standard in ECDC publications), but we used a higher level of differentiation in older people, due to the known risk factor “age” for LD. The greater regions correspond to the Nomenclature of Territorial Units for Statistics (NUTS)-2-level. Categories with most observations were chosen as reference categories, except for the seasonality (first month of the year).

We constructed two multivariable mixed-effect logistic regression models, both including the variables sex, age group, season, time trend, and test method. One model included the region and the other the laboratory as random effect. This partition was necessary due to collinearity and bias between the two variables and the outcome variable, which is shown in the results section “Regional differences” and discussed in the discussion section “Regional differences across Switzerland”.

### Ethical Statement

The study was conducted under the Epidemics Act (SR 818.101). The data, provided by laboratories, were anonymised for analysis. Other data (notification data, population statistics) are publicly available from the FOPH or the Swiss Federal Statistical Office.

The data that support the findings of this study are available from the corresponding author, DM, with the permission of the FOPH and the Federal Food Safety and Veterinary Office (FSVO), upon reasonable request.

## 3. Results

### 3.1. Data Received

The 14 laboratories provided a total of 154,851 observations, including 2808 positive tests. Three laboratories could not provide data for the entire study period (2007–2016) due to changes in their laboratory information system and data storage.

### 3.2. Exclusion

Applying pre-defined exclusion criteria (residence outside of Switzerland, inconclusive test results, tests performed outside of the study period), we excluded 6721 observations (134 positives and 968 with an inconclusive or missing test result). Additionally, 762 (13 positives) entries were excluded, for which information on either sex or age was missing.

We excluded 7287 duplicates (412 positives) from the dataset. It was further decided to exclude all serological tests due to their limited utility in a clinical/diagnostic setting (see Supplementary File 2 for details). In total 2558 (1.8%) serological tests (108 positives) were performed. Lastly, 13,196 repeated tests (383 positives) were excluded. The final dataset comprised 126,422 (1638 positives) observations.

### 3.3. National Notification System for Infectious Diseases

**Figure 1:**
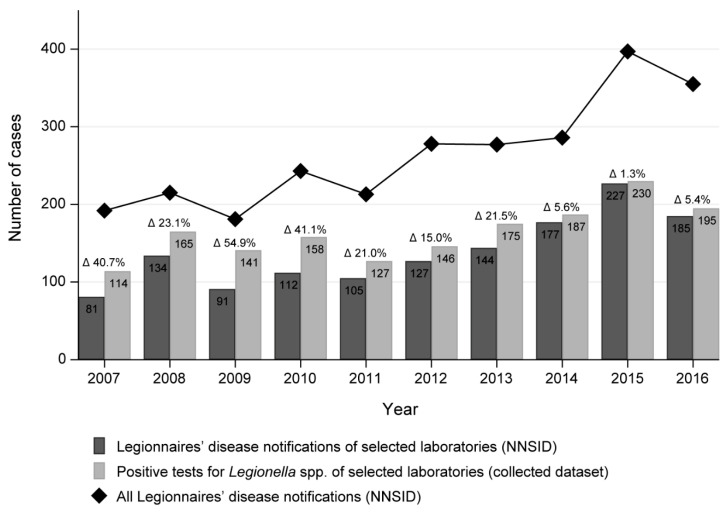
Number of Legionnaires’ disease (LD) notifications of the 14 selected laboratories as reported in the Swiss National Notification System for Infectious Diseases (NNSID) and the number of positive tests of the selected laboratories, as well as the total number of LD notifications reported in the NNSID per year, 2007–2016, Switzerland. The figures in the bars correspond to the number of observations; the relative difference between them is denoted as the percentages above the bars.

We compared the number of positive test results in our dataset to the NNSID notification numbers as notified by our selected laboratories. As noted above, the published notification numbers only reflect LD cases while the positive test results in our dataset reflect all legionellosis cases. The biggest difference in numbers was observed in 2009 with a relative difference of 54.9% (91 LD cases in the NNSID compared to 141 positive test results in our dataset; Figure 1). The average relative difference was 23.0%. Generally, the annual case number from all participating laboratories combined was higher in our dataset than in the NNSID data.

The LD cases notified to the NNSID from the 14 selected laboratories account for 54% of all notified cases nationwide between 2007 and 2016 according to the NNSID database. This proportion remained constant across the years.

### 3.4. Positivity

**Figure 2:**
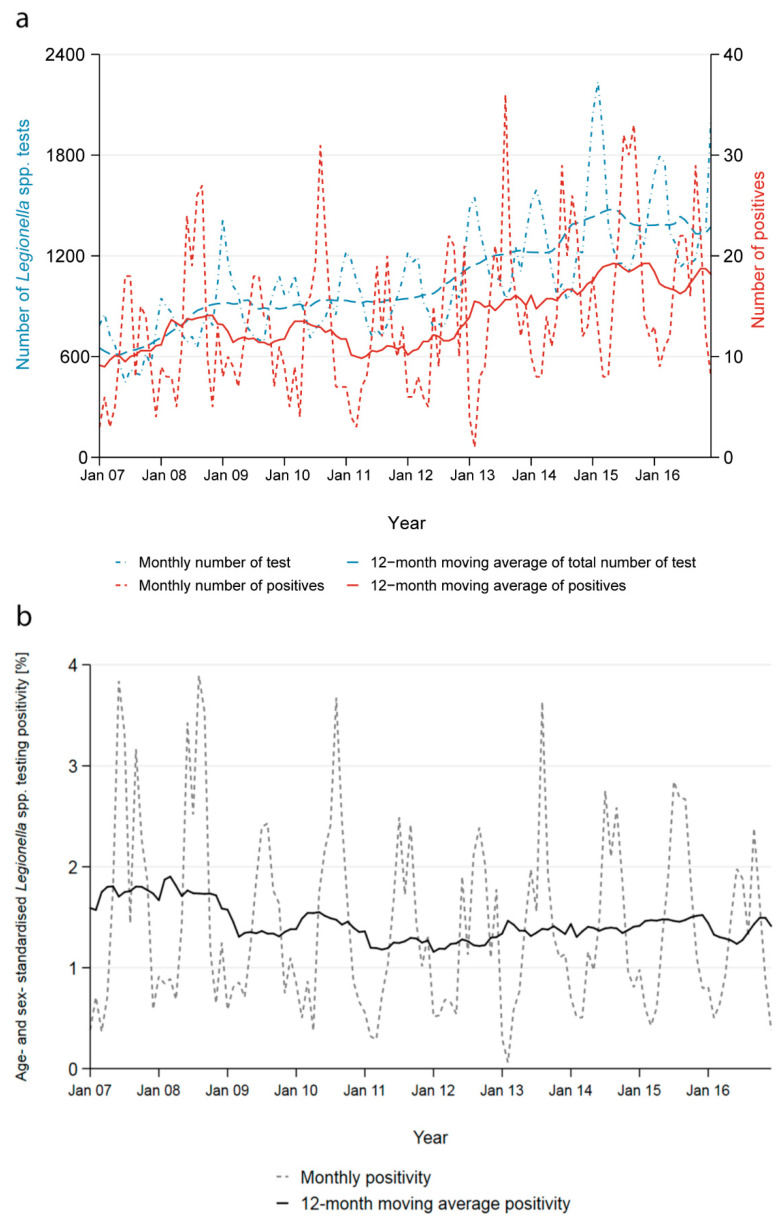
Time trend in test volume, cases and positivity. (**a**) Twelve-months moving average (solid lines) and monthly (dashed lines) number of *Legionella* spp. tests performed and number of positive tests, for the entire study period (2007–2016) by 14 diagnostic laboratories in Switzerland. (**b**) Twelve-months moving average (solid line) and monthly (dashed line) age- and sex-standardised positivity of *Legionella* spp. testing, Switzerland, 2007–2016.

The number of tests performed increased by 131% from 7366 in 2007 to 17,027 in 2016 and the number of positives by 71% from 114 to 195 (Figure 2a). The yearly age- and sex-adjusted positivity decreased marginally from 1.5% to 1.1% (Figure 2b).

Across all years, the positivity started increasing in May and peaked in August and September reaching 2.6%, then decreased in October to reach an all-year low in February with 0.5%. The seasonality of the positivity is a direct result of the contrasting seasonality of the number of tests performed and the number of positive test results obtained (Supplementary Figure S2). Most tests were performed during the winter months; on average 62% more tests were conducted in February than in August. Conversely, more than three times as many cases were reported in September compared to February.

The seasonality persisted across all age groups, both genders and all regions. It is most strongly reflected in tests performed using urinary antigens. PCR and culture-based tests do not show any clear seasonal pattern for the number of tests performed and the number of positive cases, also explained by small numbers.

### 3.5. Gender and Age

The positivity varies strongly by gender and age group. Males have an overall higher positivity compared to females (1.6% to 0.9%). The positivity increases with age and is highest among 45–64-year-olds (2.5% for males and 1.3% for females) and then decreases gradually again; this pattern is similar for both genders (Supplementary Figure S3a). The positivity of males aged 5–14 years old is the only exception to this pattern with a positivity of 1.4%. No female in the age groups “0–4” and “5–14” was tested positive.

The majority of patients tested were males (57.9%, N = 73,224). This proportion remained stable across the study period (2007–2016). The overrepresentation of males in the tested population was seen in all age categories, except in the oldest (85+ years old), where 48.7% of all tested patients were male (chi-square test: *p* < 0.01, Supplementary Figure S3b).

Overall, most tests were performed in the age group of 75–84-year-olds (25.6%, N = 32,349), closely followed by the age group of 45–64-year-olds (24.5%, N = 30,956). Least tests were performed in the age groups of infants (0–4), adolescents (5–14) and young adults (15–24) with 0.3%, 0.4% and 2.3%, respectively. During the study period, the age distribution of tested patients remained similar and the median age increased only marginally from 69 years old in 2007 to 71 in 2016 (Kruskal–Wallis test: *p* < 0.01).

The difference in sex distribution was small but statistically significant for all greater regions (range 57.5% to 60.4% males, chi-square test: *p* < 0.01) and slightly more variable between laboratories (53.3% to 64.4% males, chi-square test: *p* < 0.01). Similarly, the median age only differed marginally, but significantly between regions (range of medians 68–73 years old, Kruskal–Wallis test: *p* < 0.01) and more strongly between laboratories (range 59–74 years old, Kruskal–Wallis test: *p* < 0.01).

### 3.6. Regional Differences

Of the 14 laboratories in our dataset, 11 were hospital laboratories accounting for 86.2% (N = 109,016) of all observations included in this analysis. However, the three private laboratories may also perform diagnostics for hospitalised patients. The laboratories performed diagnostics mainly for patients with residency in proximity to the laboratory site. Therefore, the variable “laboratories” is correlated with the variable “greater region” (Supplementary Figure S4). Hence, any information on regions is heavily influenced by the selection of laboratories.

**Figure 3:**
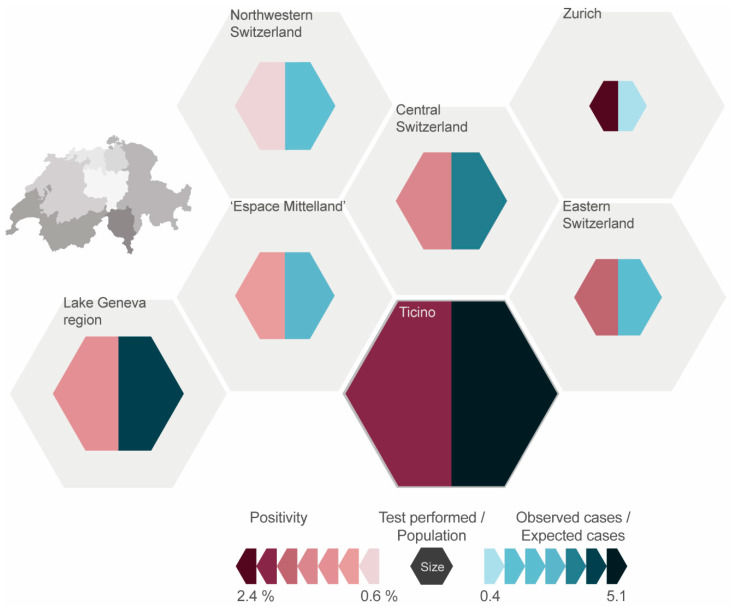
Representation of the seven greater regions of Switzerland (displayed as grey area) of the positivity for *Legionella* spp. testing (colour of left part of the smaller hexagon), the number of tests performed in relation to the resident population (size of smaller hexagon) and the ratio of the number of observed cases to expected cases (colour of right part of the smaller hexagon), based on the testing data of 14 Swiss diagnostic laboratories (2007–2016). The expected cases were calculated based on the relative population size of each region to the overall Swiss population and the proportion of each region of all cases (in our dataset).

The positivity in the greater regions across all years ranged from 0.9% in “Northwestern Switzerland” to 2.4% in the region “Zurich” (Figure 3). The positivity for all regions decreased from 2007 to 2016 except in “Northwestern Switzerland”, where there was a relative increase of 44%. The positivity fluctuates throughout the years, most notably in “Zurich” (range 1.1% to 4.5%).

Over the entire study period (2007–2016), most tests were performed in the “Lake Geneva” region, followed by “Espace Mittelland” and “Ticino”. The least amount of tests was reported from the region “Zurich”. In relation to the average population of the regions (2007–2016), much more tests were conducted in “Ticino” with 6493 tests per 100,000 population compared to “Zurich” with 377 tests per 100,000 population (Figure 3). The average for all greater regions was 2028 tests per 100,000 population.

The number of tests performed increased in all regions between 2007 and 2016 (Supplementary Figure S5). The biggest relative increase (129 in 2007 to 1698 in 2016) was observed for “Northwestern Switzerland”, followed by “Espace Mittelland” (544 to 3055) and “Eastern Switzerland” (455 to 1332). “Zurich” had the smallest relative increase (536 to 585). This distribution remained stable, even when disregarding the laboratories not providing data for the entire study period.

### 3.7. Diagnostic Methods and Sample Material

The process of exclusion of repeated tests could already provide first insights on the diagnostic procedures used in the laboratories; hence, we shortly describe the raw data set here. In the raw dataset, 7.7% (N = 10,809) of all patients were tested at least twice during the same disease episode; 4.3% (N = 6022) of the patients were tested more than once on the same day. After excluding tests performed on the same day—as the order of test could not be assessed—3.4% of all urinary antigen tests (UATs), 14.8% of all culture-based tests and 14.7% of all PCR tests were excluded as repeated tests. The positivity among the repeated tests was 2.9%.

All results henceforth stem again from the analysis of the cleaned dataset (omitting repeated tests). The positivity of *Legionella* spp. tests performed using UATs was 1.3%, using culture-based tests it was lower (0.8%) and using PCR higher (3.1%). The positivity of UATs varied based on the exact test used (Fisher’s exact test: *p* < 0.01): The UATs from lowest to highest positivity were, Binax™ Legionella Urinary Antigen EIA (Alere) (0.9%), BinaxNOW^®^ *Legionella* ICT (Alere) (1.2%), Biotest Legionella Urinary Antigen Enzyme Immunoassay (EIA, Biotest) (1.7%) and Sofia Legionella Fluorescent Immunoassay (FIA, QUIDEL) (2.2%).

The positivity of UATs decreased during the study period from 1.6% in 2007 to 1.1% in 2016. The positivity of culture-based tests remained below 1% except for three years (2008: 1.5%; 2012: 1.6%; and 2014: 1.4%). The positivity of diagnostic tests using PCR increased gradually since 2011 from 2.8% to 4.8%.

The majority of diagnostic tests performed were UATs with 90.1% (N = 113,863) followed by culture-based methods (6.6%, N = 8373) and PCR (3.3%, N = 4169). This distribution remained stable at large between 2007 and 2016. Only PCR slightly gained importance (0.8% in 2007 to 2.5% in 2016) at the costs of UATs (92.4% to 88.3%). 

UATs performed were mostly BinaxNOW (71.4%), Binax (11%), Biotest (10.1%) and Sofia Legionella FIA (7.6%). The Sofia Legionella FIA test was introduced only in 2014 and increased its market share since to 28.6% of all UATs in 2016. For BinaxNOW market shares decreased from 75.7% of all UATs in 2007 to 52.3% in 2016.

Almost all of the nine laboratories performing PCR used a different type of test. Four laboratories reported to outsource PCR diagnostics to other laboratories and, therefore, could not provide detailed information. Three laboratories had communicated to use respiratory multiplex PCR panels; however, for two of three, the distinction between single and multiplex PCR could not be made in our dataset (personal communication, May–July 2017). Due to this heterogeneity and lack of accuracy, we did not further quantify the different types of PCR tests performed.

As the test method is dependent on the laboratories and their diagnostic procedures, the variable “method” is correlated with the variable “laboratory” and therefore also with “region” (see Supplementary Figure S4).

Twelve of the 14 laboratories predominantly or exclusively performed UATs. One laboratory performed 76.3% PCRs and another 79.3% culture-based diagnostics. The proportion of PCR increased in the former between 2007 and 2016 replacing UATs, while in the latter the proportion of culture-based tests and UATs increased replacing PCR.

UATs comprise at least 80.6% of all tests performed in all greater regions. The biggest proportion of culture-based tests was performed in “Espace Mittelland” (11.5%), “Lake Geneva region” (9.8%) and “Northwestern Switzerland” (8.9%). Most PCR tests were performed in “Northwestern Switzerland” (10.5%) and “Lake Geneva region” (3.2%). In four of the seven regions, the diagnostic methods used over the years remained overall unchanged (Ticino, Central Switzerland, Eastern Switzerland, Zurich).

### 3.8. Determinants for a Positive Test Result of Legionella spp.

The univariable model showed a significantly increased odds ratio (OR) for a positive test outcome for the test years 2007 and 2008 compared to the latest test year 2016. The time trend variable showed a marginal downward trend, with a rounded OR of 1 (exact OR 0.998, CI 0.9970–0.9998, *p* = 0.03). Further, all calendar months from May to December had significantly increased odds for a positive test outcome compared to February. The highest odds were calculated for August and September (OR 4.02, *p* < 0.01 for both).

Females were almost half as likely as males to be tested positive for a *Legionella* spp. infection (OR 0.56, *p* < 0.01). Compared to the reference group of 75–84 year olds, the age groups “15–24” and “85+” had significantly decreased odds for a positive test outcome (OR 0.41, *p* < 0.01 and OR 0.76, *p* < 0.01), and the age groups “45–64” and “65–74” showed increased odds (OR 2.06, *p* < 0.01 and OR 1.36, *p* < 0.01).

“Northwestern Switzerland” showed 20% lower probability for a positive test result compared to the “Lake Geneva” region (OR 0.81, *p* = 0.04), while “Zurich” had more than double the odds and “Ticino” a 50% increased chance for a positive test result (OR 2.22, *p* < 0.01 and OR 1.47, *p* < 0.01).

Culture-based tests had lower odds for a positive test outcome compared to UATs (OR 0.63, *p* < 0.01). In contrast, PCR tests had 2.5-fold increased odds for a positive test (OR 2.47, *p* < 0.01).

The univariable regression using the sample material as an explanatory variable was stratified by culture-based tests and PCR. For culture-based tests, using material obtained through paracentesis or using sputum showed the highest OR (OR 10.32, *p* = 0.03 and OR 5.12, *p* < 0.01). Using PCR, material obtained through paracentesis (OR 4.47, *p* = 0.05) or swabs (OR 3.91, *p* < 0.01) or using sputum (OR 3.24, *p* < 0.01) had elevated odds for a positive test outcome.

**Figure 4:**
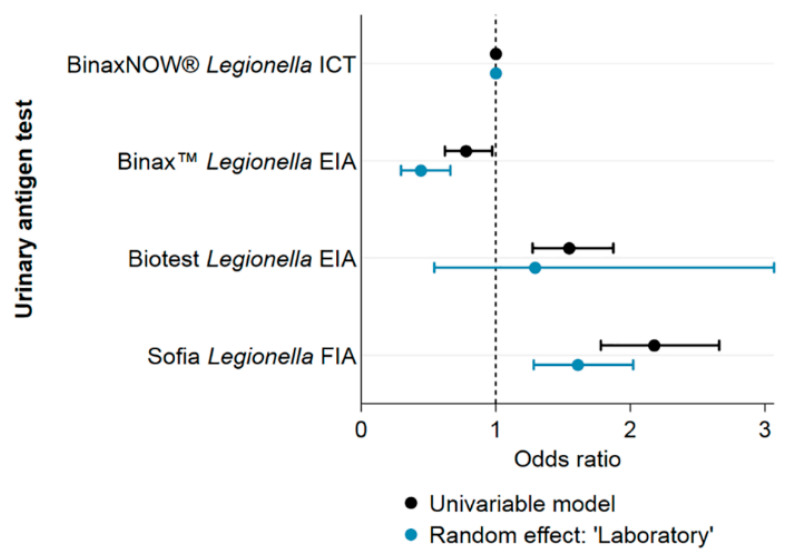
Differences in positivity across UAT test kits. Univariable regression results with and without random effect on “laboratory” for the outcome of having a positive test result for *Legionella* spp. in Switzerland, 2007–2016.

Figure 4 shows the ORs for different UATs before and after inclusion of “laboratory” as a random effect. Univariable models including other variables showed no significant effect on the ORs and are therefore not shown.

**Figure 5:**
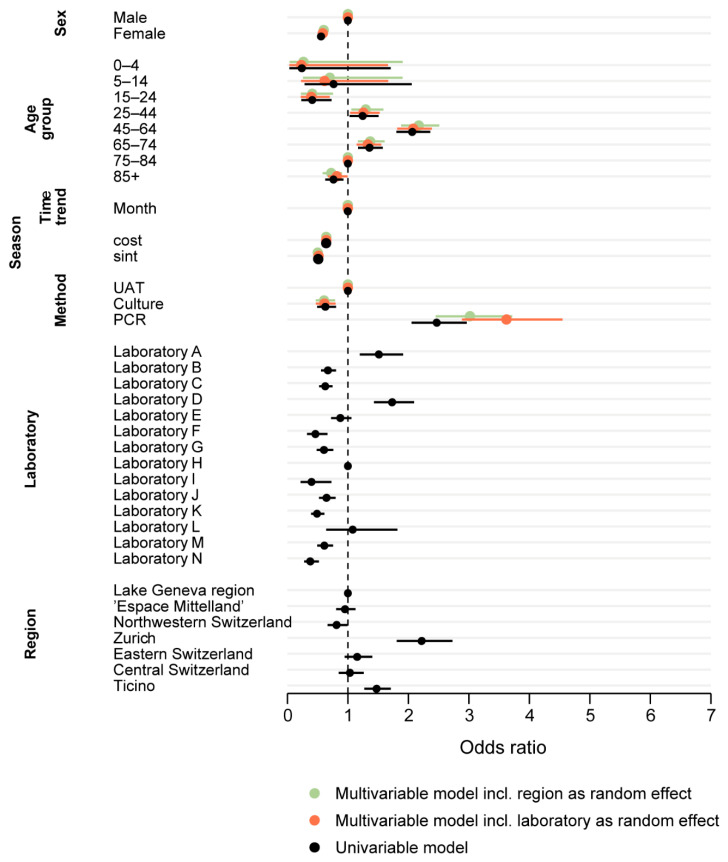
Determinants for a positive test result for *Legionella* spp. Multivariable mixed-effect logistic regression compared with the univariable regression results for the outcome of having a positive test result for *Legionella* spp. in Switzerland, 2007–2016.

Both multivariable mixed-effect logistic regression models (with the inclusion of “region” or “laboratory” as random effect, respectively) are shown in Figure 5 together with the results of the univariable models. The estimates are comparable for all variables. The marginal but statistically significant negative OR for the time trend-variable, however, lost its statistical significance in both multivariable models.

## 4. Discussion

We collected the testing data of 14 Swiss diagnostic laboratories and calculated the positivity, i.e., the proportion in the number of positive tests to the number of tests performed to investigate the increase observed in case numbers of LD in official disease surveillance.

### 4.1. Time Trend in Positivity 2007–2016

The number of *Legionella* spp. tests performed increased more strongly than the number of cases found, resulting in a marginally decreasing positivity between 2007 and 2016 from 1.5% to 1.1%. However, no temporal trend was found in the multivariable regression models. The strong increase in test numbers for *Legionella* spp. cannot be explained given that contextual information on health-seeking, test behavior of physician and on diagnostic methods and procedures applied by laboratories are essential for a correct interpretation of trends in positivity.

We hypothesise that changes in the diagnostic methods influenced the number of tests performed: Especially, the introduction of the UAT revolutionised the diagnosis of legionellosis. In 2015, 78.2% of all LD cases in Europe were detected using UATs [20]. In Switzerland, UATs were introduced to the routine diagnostic in 1997 and are now predominantly used [21]. However, the UAT is unlikely to have influenced the most recent increase in test numbers, as the introduction of this test occurred almost 20 years ago and the proportion of UATs performed remained stable or declined during the study period. Therefore, changes in testing behaviour of physicians, health-seeking behaviour of patients, prevalence of risk factors and of disease frequency need to be considered to explain the increase in test volume.

Symptom-based testing explains the inverse seasonality in the number of tests performed and the number of cases found. Community-acquired pneumonia (CAP) peaks during the winter months but is predominantly caused by agents other than *Legionella* spp. [22]. Therefore, the testing volume is higher in winter than in summer even if the physicians are aware of a summer peak for *Legionella* spp. [23].

The Swiss Society of Infectious Diseases (SSI) provides guidelines for the management of CAP, which were adapted from European guidelines. The guidelines state that microbial testing is not indicated in primary care settings, and even in hospital settings, *Legionella* spp. testing may only be useful for selected risk patients based on clinical or epidemiological features [24]. These recommendations leave room for interpretation and can, thus, be applied differently by the treating physician, depending on his/her awareness of LD and knowledge of its epidemiological and clinical features. Failure to diagnose LD has previously been attributed to a lack of awareness of LD [25]. Heightened awareness of physicians and consideration of LD in their differential diagnosis of patients presenting with pneumonia would lead to more LD tests ordered over time. However, there is a lack of information on adherence to the CAP guidelines, the awareness level among Swiss physicians and the case management of LD in Switzerland.

An increasing number of patients in Switzerland seek care for non-urgent or non-life-threatening conditions at emergency departments rather than at primary care level [26]. These “new” patients presenting at the emergency department could contribute to a higher number of LD tests conducted: According to SSI guidelines, microbiological investigation of pneumonia is recommended earlier in the hospital compared to the primary care setting. Increased awareness and change in health-seeking behaviour as potential causes for increased testing are independent of disease incidence, but rather represent a shift in test practices. More cases are found if a larger part of the population is being screened for LD, hence, reducing the extent of underestimation.

However, an important alternative explanation for the increase in test volume is that, actually, more ill patients present with signs and symptoms of LD (i.e., pneumonia). In this scenario, the increase in test numbers would be explained by an increase in incidence rather than a decrease in the extent of underestimation. According to the “medical statistic of hospitals” (“Medizinische Statistik der Krankenhäuser”) published annually by the Federal Statistical Office, the number of hospitalised patients with pneumonia recorded as “main diagnosis” in over 14-year-olds has increased by one third between 2007 and 2016, while the number decreased for patients younger than 15 [26]. Hence, these statistics support the hypothesis of increased pneumonia incidence leading to higher test volumes.

However, the lack of information and understanding of the trajectory from health-seeking to LD diagnosis does not allow conclusive interpretation of the 10-year-trend in positivity. The number of reported LD cases is not only rising in Switzerland but also in the EU/EEA countries, which are members of the European Legionnaires’ Disease Surveillance Network (ELDSNet) and in the US [4,27]. Nevertheless, Switzerland had the highest notification rate per 100,000 population in 2017 (5.8), followed by Slovenia (5.7), Denmark (4.8) and Italy (3.3) and the second largest increase in notification rate between 2013 and 2017 [4,28]. However, we are not aware that data from these national surveillance systems were evaluated considering denominator data. Furthermore, notification rates are heavily influenced by the health system itself, and hence, comparability between countries is limited.

### 4.2. Male and Elderly People at Risk

We found that men were more often tested for *Legionella* spp. than women were, and positivity was significantly higher for males than for females. This suggests that the higher case numbers for men are actually due to a higher incidence in the male population or a diverging health-seeking behaviour rather than more thorough testing due to male sex being a known risk factor [29].

Regardless of gender, adults over 25 years showed an increased positivity, peaking at 45–64 years of age and declining again in older age groups. The difference in positivity for the middle-aged patients (25–64) compared to the older patients (over 65) is likely due to a more thorough testing approach for the older patients compared to younger patients (testing elderly earlier, presenting with less severe acute respiratory infections). The difference in test volume could also be due to the knowledge that older age is a risk factor for LD or CAP being more prevalent in older age [6,8,30].

### 4.3. Regional Differences Across Switzerland

It is difficult to estimate regional differences based on our data, as it is heavily dependent on the laboratories included in the study. The interaction of these variables (“laboratory” and “greater region”) is not straightforward to assess. Apart from the collinearity of these variables, we found that the positivity of tests performed in one laboratory differed substantially depending on the residency of the patient, especially if the patient lived outside of the usual catchment area of said laboratory. We assume this is due to different pre-test probabilities for a positive test outcome, e.g., only the samples of immunosuppressed patients (with an assumed higher probability of an actual infection with *Legionella* spp.) would be sent to another laboratory for confirmation. However, it is impossible to control our dataset for these “outsourced” tests. For this reason, we decided to construct two multivariable logistic regression models. These limitations should be kept in mind when interpreting the results on the greater regions.

The calculated positivity and the logistic regression models show heterogeneity across regions. The regions of “Zurich” and “Ticino” seem to identify more positive cases per number of tests performed. Additionally, the two regions show opposite test frequency: relative to the resident population, in “Ticino” many people are tested for LD while the inverse applies for “Zurich”. Further, “Ticino” has the highest notification rate amongst all Swiss cantons, which might not only result from the highest testing volume but from an actually increased incidence, as indicated by the increased positivity and suggested by other studies focusing on the impact of climate at regional level (2018) [12]. In contrast, in “Zurich”, where the number of reported cases and case rate is similar to the national average (2008–2017), either testing is more targeted to the “correct” patients, resulting in a higher positivity or incidence is actually higher, but underestimated due to the small test number [6]. In “Northwestern Switzerland” the reporting rate is also on the national average, but the region has significantly decreased odds for a positive test outcome. The number of tests performed increased most strongly in this region. At the same time, it was the only region with an increasing positivity.

As has been mentioned before, the SSI guidelines are subject to the interpretation of the health personnel, and the level of adherence is unknown. Hence, who is tested is likely very heterogeneous across Switzerland.

### 4.4. Heterogeneity in the Diagnostic Methods

Not all diagnostic test methods for *Legionella* detect the same pathogens and strains. The application of UAT is limited mainly to *Legionella pneumophila* serogroup 1, culture-based techniques can detect all *Legionella* species, while PCR techniques can either detect only *L. pneumophila* or all species, depending on the type of test. The positivity also varies depending on the test method used, on the application of the chosen test method and on the specific kind of test kit or manufacturer.

Using PCR increased the odds of obtaining a positive test result significantly compared to UAT and cultures. This could be attributed to the higher sensitivity of PCR compared to UAT, but it could also result from false-positives due to cross-reaction or contamination [31]. A systematic review comparing UAT and PCR found PCR to be preferable. However, PCR is limited by the availability of appropriate sample material from the patient [32]. Culture is still regarded as the gold standard, as it allows the cultivation of strains but exhibits an overall lower sensitivity, which corroborates with our results [31]. However, in our study, the differences in positivity between UAT, PCR and culture could also be attributed to different testing behaviours, rather than to features inherent to the test. In some hospitals/laboratories, PCR and culture-based tests may only be performed in high-risk patients (e.g., immunocompromised patients), which might affect the positivity and introduce bias.

Double-testing with different diagnostic methods is often seen as advantageous [32,33,34]. The European Study Group of *Legionella* Infections (ESGLI) further recommends that all samples positive by UAT should be retested after heat treatment of the urine for confirmation unless the initial sample was already boiled [35,36]. However, in our raw dataset, only 1 in 12 patients got tested at least twice during the same disease episode. UATs are most often used as a stand-alone test, and only in 194 cases, a positive UAT was repeated during the same disease episode. However, it is likely that not all secondary tests for confirmation are registered in the individual laboratory information systems and, hence, are possibly not reported.

Lastly, the choice of test kit manufacturer for the widely used UAT also influences the positivity of *Legionella* spp. testing: the Sofia *Legionella* FIA test has a significantly increased positivity compared to the most commonly used UAT BinaxNOW. In comparison, the former has a higher sensitivity but also a lower specificity especially without heat treatment of the urine, which could lead to false-positive results [37]. The Swiss national reference centre for *Legionella* (NRCL) recommends heat treatment of all urine samples if Sofia *Legionella* FIA is used. If the initial urine sample was not boiled, a confirmation test needs to be performed on positive samples. However, it is unclear how many laboratories adhere to these recommendations of the NRCL. Hence, differences in positivity of the various test kits might not only be inherent to the kit itself but also to the performance of the test. It should be noted that due to the correlation of the test method (kit) and region, these differences could also be impacted by differences in regional incidence. However, the calculated positivities and the distribution of methods do not all point in the same direction and performance differences of test kits have been demonstrated before.

It is evident that diagnostic test practices, including patient selection for testing, choice of test method and the performance of the diagnostic test influence test outcomes. From our dataset, heterogeneity between preferred diagnostic test methods is observed. There is a high degree of uncertainty linked to physicians’ testing behaviours and also test performance in the laboratories, or rather the physicians’ adherence to existing guidelines. An assessment of current practices and the harmonisation across Switzerland could improve public health surveillance and decrease heterogeneity (e.g., of levels of underestimation) between regions.

### 4.5. Limitations

For feasibility reasons, considering over 106 laboratories are either authorised or accredited in Switzerland [38], the analysis had to be limited to a selection of laboratories. We chose to base the selection on the volume of notifications during the study period, therefore, favouring laboratories with the highest notification rates. With the 14 selected laboratories, 54% of all notifications between 2007 and 2016 could be covered. Detailed information on the selected laboratories, e.g., laboratory coverage, testing volume by greater region and laboratory identifiers, are in parts restricted for data confidentiality reasons.

## 5. Conclusions

We found a stable positivity for *Legionella* spp. testing between 2007 and 2016 analysing the testing data of 14 Swiss diagnostic laboratories. There is a proportional increase in the number of cases identified in relation to the number of diagnostic tests performed. However, it is not clear why the number of tests performed more than doubled in the 10-year study period. The interpretation of the positivity curve and the implications on disease incidence can be vastly different depending on the reason for the increase in testing volume.

The assessment is further complicated, as large variations in positivity and test volume across the seven greater regions of Switzerland exist. We assume that these differences are only partly explained by differences in actual disease incidence; they seem to also stem from different CAP case management and diagnosis plans and represent different degrees of underestimation. The scarcity of data impedes evaluation of the different hypotheses. The diagnostic method greatly influences the test outcome. Culture-based methods, PCR and UATs perform differently and have their own limitations; particularly as in the case of the latter compliance with the recommendations and standard operating procedure for boiling of the urine are suspected to vary.

The lack of national (or adherence to existing) guidelines and the heterogeneity of the diagnostic tests and testing procedures applied hampers the diagnosis of LD as well as comparison of data in a public health context. Therefore, diagnostic procedures should be harmonised across Switzerland to follow recommendations from the national reference centre for *Legionella*.

## Figures and Tables

**Table 1:** Overview of the variables used in the regression models on a positive test result for *Legionella* spp. in Switzerland, 2007–2016.

Variable	Format	Content
Age group	Categorical	0–4, 5–14, 15–24, 25–44, 45–64, 65–74, 75–84, 85+ years old
Greater region	Categorical	Lake Geneva region, “Espace Mittelland”, Northwestern Switzerland, Zurich, Eastern Switzerland, Central Switzerland, Ticino
Laboratory	Categorical	14 selected Swiss diagnostic laboratories (11 hospital-associated and 3 private)
Method	Categorical	PCR, UAT, culture
Sample material	Categorical	Bronchial-liquid, urine, blood, biopsy, sputum, swab, paracentesis, liquid, other
Season	Numeric (float)	sin((d × 2 × π)/T) and cos((d × 2 × π)/T); d = time period (e.g., January, February), T = number of time periods (e.g., 12 months)
Sex	Binary	Male, female
Test result	Binary	Negative, positive
Time trend	Numeric (float)	Combination of month and year, e.g., January 2007 = 1, February 2007 = 2, February 2008 = 14

## Supplementary Materials

The following are available online at https://www.mdpi.com/1660-4601/17/19/7343/s1, Figure S1: Different example scenarios based on the definition of disease episode to exclude repeated tests for *Legionella* spp. in Switzerland, 2007–2016. Text file S2: Descriptive analysis of the serological tests performed for *Legionella* spp. in Switzerland (2007–2016) as provided in the raw dataset by 14 Swiss laboratories and argumentation for their exclusion for further analysis. Figure S2: Seasonality in test volume and cases. The average and interquartile range (IQR) per calendar month of the total number of *Legionella* spp. tests and number of positive tests, Switzerland, 2007–2016. The seasonality has been incorporated into the mixed effect logistic regression using sine and cosine functions, in the form of sin((d × 2 × π)/T) and cos((d × 2 × π)/T), where d is the time period (e.g. January, February) and T is the number of time periods (e.g. 12 months), as described by Stolwijk, A. M., et al. (1999). Figure S3: Age distribution in test volume and positivity (a) Positivity of *Legionella* spp. testing by sex and age groups, Switzerland, 2007–2016. (b) Number of *Legionella* spp. tests performed by sex and age groups in Switzerland (2007–2016) and permanent resident population in Switzerland (2016) by sex and age groups. Figure S4: Correlation between the variables “greater region” and “laboratory” included in the *Legionella* spp. positivity study, Switzerland, 2007–2016. Figure S5: Trends of the total number of tests performed per greater region by 14 diagnostic laboratories included in the *Legionella* spp. positivity study, Switzerland, 2007–2016. References [39,40,41,42,43,44,45,46,47,48,49,50] are cited in the supplementary materials.
