# Supplementary material for: Legionnaires’ Disease on the Rise in Switzerland: A Denominator-Based Analysis of National Diagnostic Data, 2007–2016
Source: Int J Environ Res Public Health. 2020 Oct 8;17(19):7343. doi: 10.3390/ijerph17197343 (PMC7579383; doi:10.3390/ijerph17197343)
Supplement: Supplementary file 1 [file ijerph-17-07343-s001.zip › PUB-Legio_suppl1_repeated_tests_v2.pdf]

Legionnaires' disease on the rise in Switzerland: A denominator-based analysis of national diagnostic data, 2007-2016

Fabienne B. Fischer[1,2], Claudia Schmutz[1,2], Valeria Gaia[3], Daniel Mäusezahl[1,2,*]

[1] Swiss Tropical and Public Health Institute, Basel, Switzerland
[2] University of Basel, Basel, Switzerland
[3] National Reference Center for Legionella, Service of microbiology, Ente Ospedaliero Cantonale, Bellinzona, Switzerland
* Author for correspondence: Dr Daniel Mäusezahl; E-mail: daniel.maeusezahl@unibas.ch; Tel.: +41-61-284-8178

SUPPLEMENTARY MATERIAL: REPEATED TESTS

To calculate the positivity, i.e. the proportion of all positive tests among all tests performed, as a proxy for the incidence rate across the years, it is essential to limit the number of tests to one for each patient and disease episode. Otherwise, if one *Legionella*-infected patient is tested multiple times, the numerator will be inflated and skews the proportion.

Patients were identified by their identification number (given by the laboratory), sex and birthdate. The disease episode was defined as one single disease event from infection to curation/death. If a patient was re-infected at any later time point, this was counted as a second disease episode. However, as the dataset investigated is limited to laboratory data and lacks clinical information, the definition of a disease episode within our dataset was rather complex.

After reviewing the literature and consultation with an expert, we made several assumptions on timeframes: i) The duration of symptoms or hospitalisation is 6-10 days [39, 40]; ii) The duration of therapy and the possibility for a relapse is 28 days [41-43]; iii) The bacteria is detectable in any given test up to 67 days [31,44-49]. Those timeframes were then anchored to the only available information we have: diagnostic tests (positive and negative) using another set of assumptions: i) Each test indicates that the patient must have symptoms, otherwise no test would be ordered; ii) Each positive test indicates that (parts of) *Legionella* were found, hence, there is a possibility for a future relapse and the detection period of the test has to be considered.

Based on these assumptions, we constructed several scenarios, on which we based the exclusion of repeated tests, some examples are shown in Figure 1. In scenario A, the second (positive test) will be excluded, as the positive test could also result from continued detection of the pathogen causing the initial infection. In scenario B, the second (negative) test is excluded, as it is within the treatment period and assumed to be control of treatment. In scenario C, the second (negative) test is excluded for the same reason as in B (control of treatment); the third (positive) test is assumed to be a new disease episode, due to the previous negative test. In scenario D, it is assumed that the indication for testing (i.e. symptoms) are independent for both tests, hence represent two disease episodes. Therefore, both tests remain in the data set.

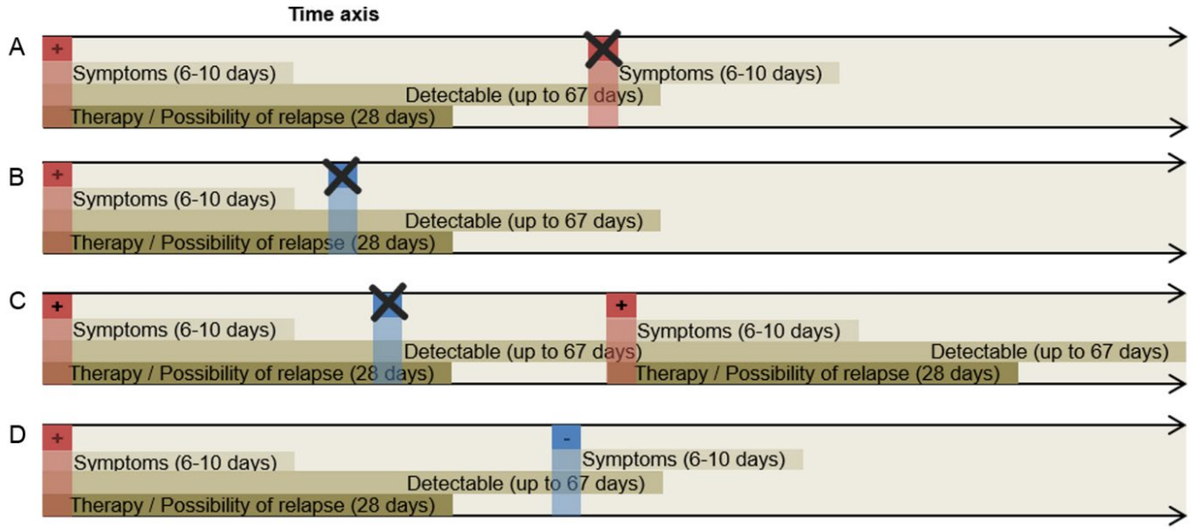

Supplementary Figure 1 Different example scenarios based on the definition of disease episode to exclude repeated tests for *Legionella* spp. in Switzerland, 2007-2016.

To avoid random exclusion of tests using different diagnostic methods for the same patients on the same day, we ordered the test methods by the total number of tests performed (i.e. urinary antigen test [UAT], culture, PCR).

The results from the exclusion based on these scenarios have been selectively and manually tested for plausibility. A sensitivity analysis has been performed alternating the timeframes, as well as the order of exclusion by test method to check the robustness of the results. The number of excluded positive and negative tests proofed to be stable.

REFERENCES

31. Peci A, Winter AL, Gubbay JB. Evaluation and comparison of multiple test methods, including real-time PCR, for Legionella detection in clinical specimens. Front Public Health. 2016;4:175. doi:10.3389/fpubh.2016.00175.

39. Pedro-Botet L, Yu VL. Legionella: macrolides or quinolones? Clin Microbiol Infect. 2006;12 Suppl 3:25-30. doi:10.1111/j.1469-0691.2006.01394.x.

40. Amsden GW. Treatment of Legionnaires’ Disease. Drugs. 2005;65(5):605-14. doi:10.2165/00003495-200565050-00003.

41. Steele RW, Bragg L. Legionella Infection Treatment & Management. 2016. Accessed 07 18 2017 2017.

42. Leverstein-van Hall MA, Verbon A, Huisman MV, Kuijper EJ, Dankert JJCid. Reinfection with Legionella pneumophila documented by pulsed-field gel electrophoresis. 1994;19(6):1147-9.

43. Sanders KL. Relapse of Legionnaires' Disease in a Renal Transplant Recipient. Archives of Internal Medicine. 1980;140(6):833. doi:10.1001/archinte.1980.00330180107030.

44. Kohler RB, Winn WC, Jr., Wheat LJ. Onset and duration of urinary antigen excretion in Legionnaires disease. J Clin Microbiol. 1984;20(4):605-7.

45. Diederen BM. Legionella spp. and Legionnaires' disease. J Infect. 2008;56(1):1-12. doi:10.1016/j.jinf.2007.09.010.

46. Sopena N, Sabria M, Pedro-Botet ML, Reynaga E, Garcia-Nunez M, Dominguez J et al. Factors related to persistence of Legionella urinary antigen excretion in patients with legionnaires' disease. Eur J Clin Microbiol Infect Dis. 2002;21(12):845-8. doi:10.1007/s10096-002-0839-5.

47. Harrison TG, Taylor AG. Timing of seroconversion in Legionnaires' disease. Lancet. 1988;2(8614):795. doi:10.1016/S0140-6736(88)92442-7.

48. Waterer GW, Baselski VS, Wunderink RG. Legionella and community-acquired pneumonia: a review of current diagnostic tests from a clinician's viewpoint. Am J Med. 2001;110(1):41-8. doi:10.1016/S0002-9343(00)00624-0.

49. Delgado-Viscogliosi P, Solignac L, Delattre JM. Viability PCR, a culture-independent method for rapid and selective quantification of viable Legionella pneumophila cells in environmental water samples. Appl Environ Microbiol. 2009;75(11):3502-12. doi:10.1128/AEM.02878-08.
