# Supplementary material for: Legionnaires’ Disease on the Rise in Switzerland: A Denominator-Based Analysis of National Diagnostic Data, 2007–2016
Source: Int J Environ Res Public Health. 2020 Oct 8;17(19):7343. doi: 10.3390/ijerph17197343 (PMC7579383; doi:10.3390/ijerph17197343)
Supplement: Supplementary file 1 [file ijerph-17-07343-s001.zip › PUB-Legio_suppl3_additional_figures_v2.pdf]

Legionnaires' disease on the rise in Switzerland: A denominator-based analysis of national diagnostic data, 2007-2016

Fabienne B. Fischer[1,2], Claudia Schmutz[1,2], Valeria Gaia[3], Daniel Mäusezahl[1,2,*]

[1] Swiss Tropical and Public Health Institute, Basel, Switzerland
[2] University of Basel, Basel, Switzerland
[3] National Reference Center for Legionella, Service of microbiology, Ente Ospedaliero Cantonale, Bellinzona, Switzerland
* Author for correspondence: Dr Daniel Mäusezahl; E-mail: daniel.maeusezahl@unibas.ch; Tel.: +41 61 284 8178

SUPPLEMENTARY MATERIAL: ADDITIONAL FIGURES

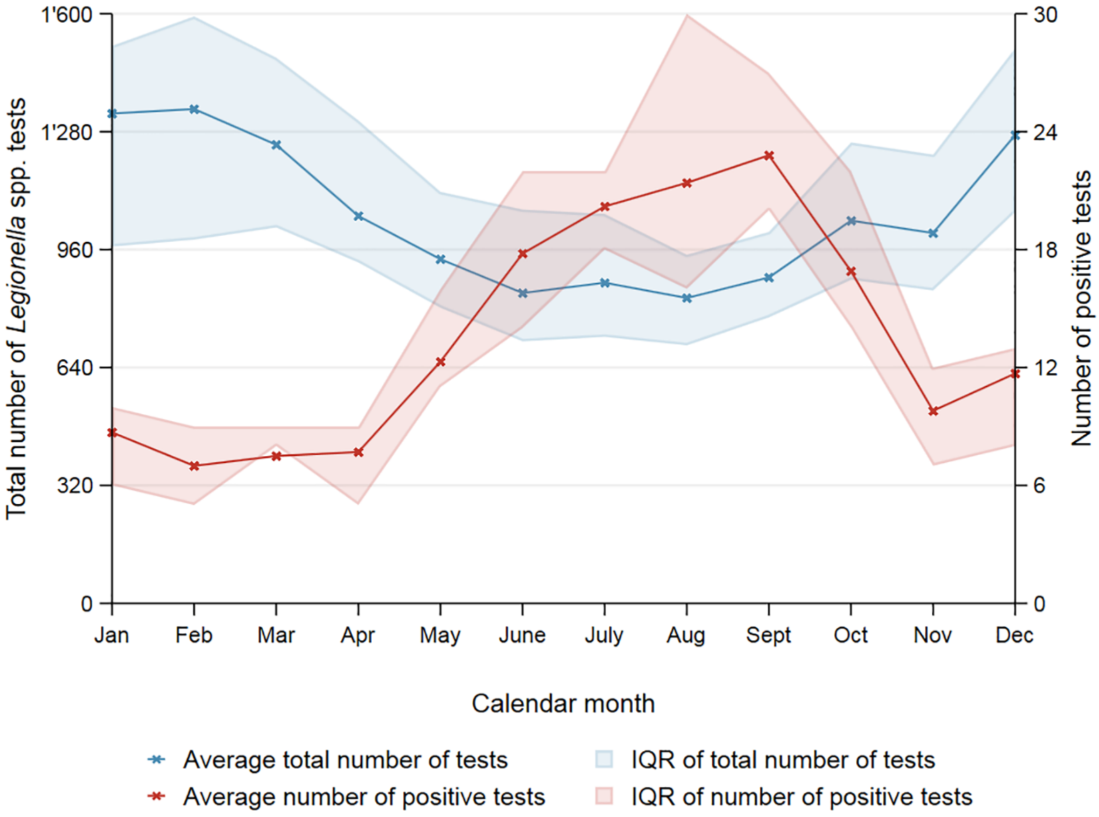

Supplementary Figure 2 Seasonality in test volume and cases. The average and interquartile range (IQR) per calendar month of the total number of *Legionella* spp. tests and number of positive tests, Switzerland, 2007-2016. The seasonality has been incorporated into the mixed effect logistic regression using sine and cosine functions, in the form of sin((d*2*π)/T) and cos((d*2*π)/T), where d is the time period (e.g. January, February) and T is the number of time periods (e.g. 12 months), as described by Stolwijk, A. M., et al. (1999).

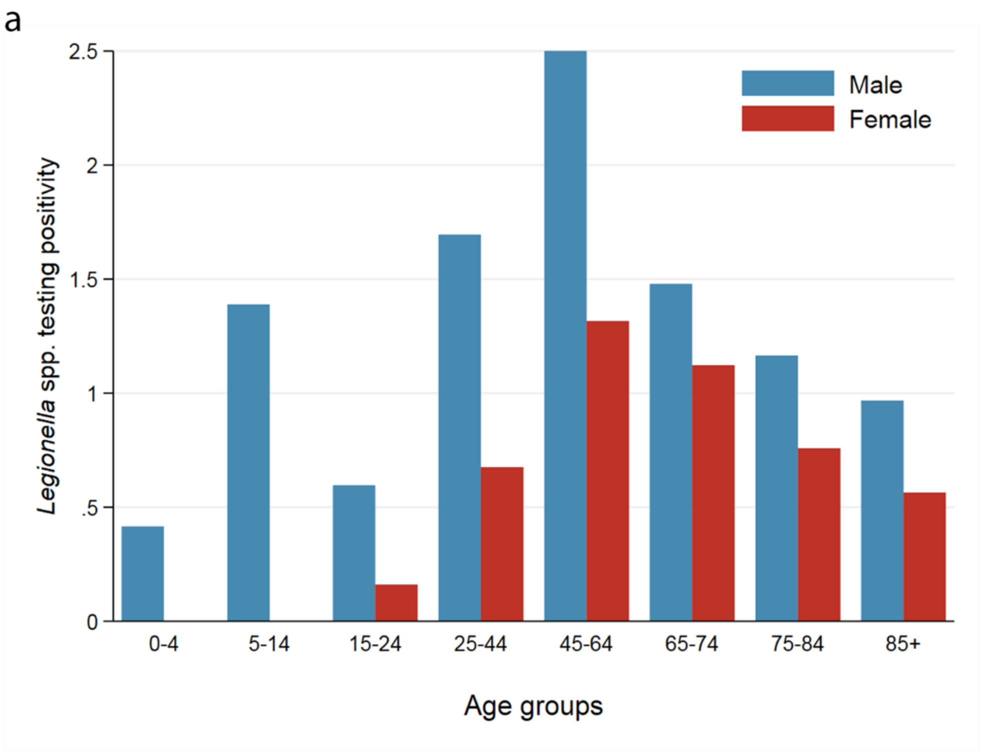

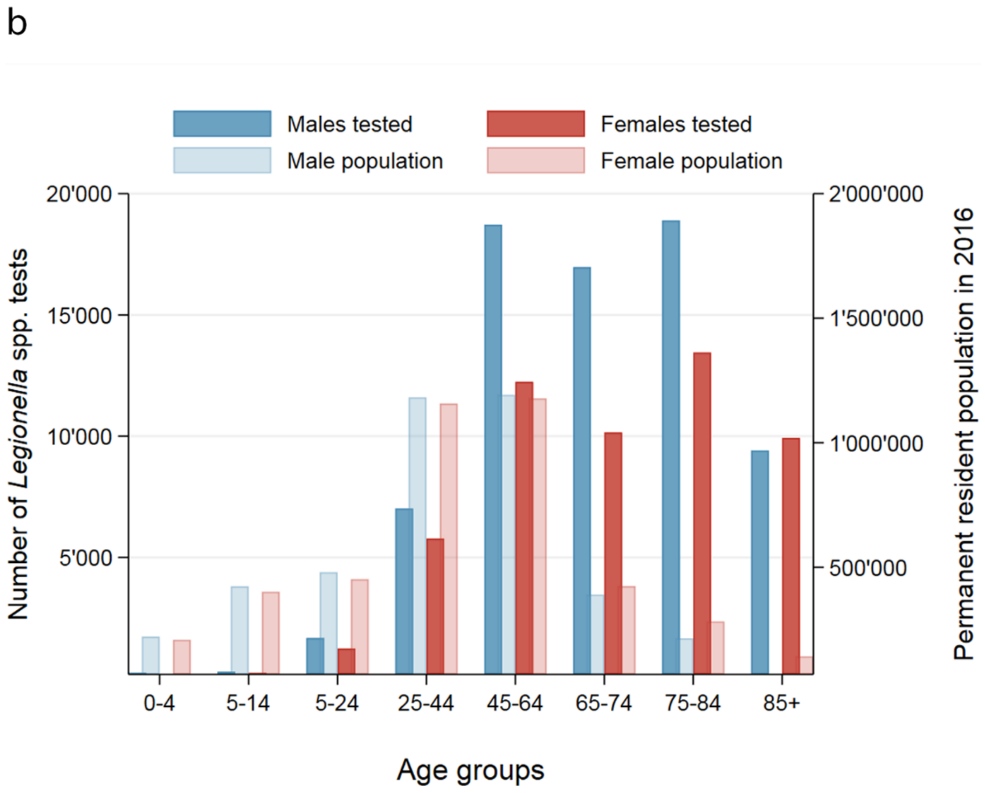

Supplementary Figure 3 Age distribution in test volume and positivity (a) Positivity of *Legionella* spp. testing by sex and age groups, Switzerland, 2007-2016. (b) Number of *Legionella* spp. tests performed by sex and age groups in Switzerland (2007-2016) and permanent resident population in Switzerland (2016) by sex and age groups.

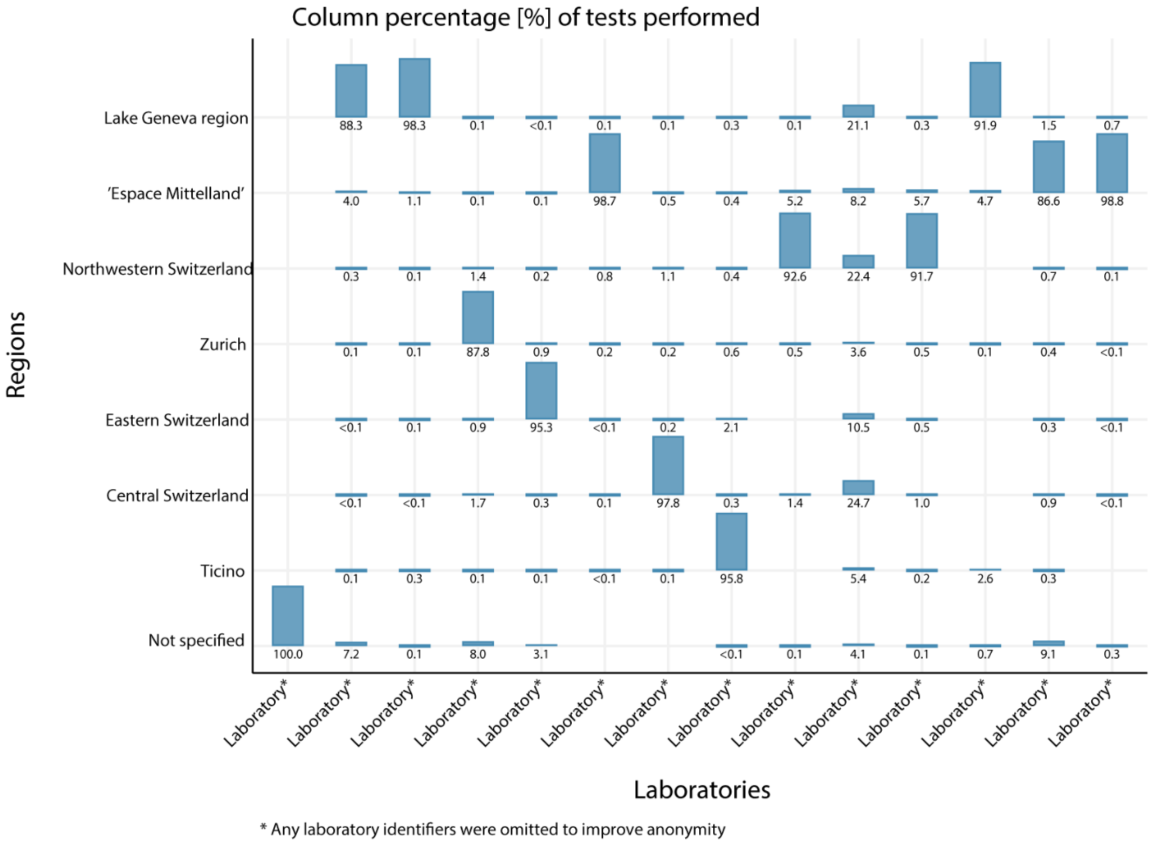

Supplementary Figure 4 Correlation between the variables "greater region" and "laboratory" included in the *Legionella* spp. positivity study, Switzerland, 2007-2016.

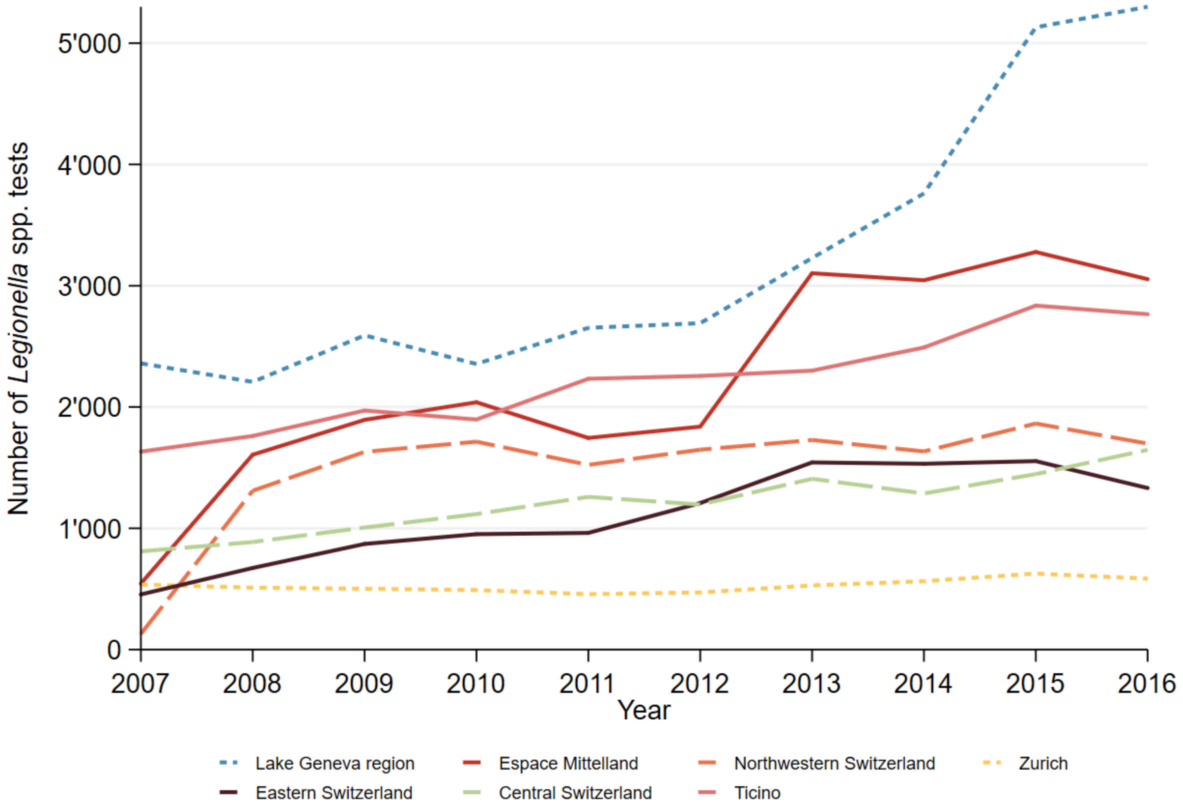

Supplementary Figure 5 Trends of the total number of tests performed per greater region by 14 diagnostic laboratories included in the *Legionella* spp. positivity study, Switzerland, 2007-2016
