# Supplementary material for: Legionnaires’ Disease on the Rise in Switzerland: A Denominator-Based Analysis of National Diagnostic Data, 2007–2016
Source: Int J Environ Res Public Health. 2020 Oct 8;17(19):7343. doi: 10.3390/ijerph17197343 (PMC7579383; doi:10.3390/ijerph17197343)
Supplement: Supplementary file 1 [file ijerph-17-07343-s001.zip › PUB-Legio_suppl2_serology_v3-FINAL-from 2nd proofreading received.pdf]

Legionnaires' disease on the rise in Switzerland: A denominator-based analysis of national diagnostic data, 2007-2016

Fabienne B. Fischer[1,2], Claudia Schmutz[1,2], Valeria Gaia[3], Daniel Mäusezahl[1,2,*]

[1] Swiss Tropical and Public Health Institute, Basel, Switzerland
[2] University of Basel, Basel, Switzerland
[3] National Reference Center for Legionella, Service of microbiology, Ente Ospedaliero Cantonale, Bellinzona, Switzerland
* Author for correspondence: Dr Daniel Mäusezahl; E-mail: daniel.maeusezahl@unibas.ch; Tel.: +41-61-284-8178

SUPPLEMENTARY MATERIAL: SEROLOGY

The initially cleaned dataset contained 2558 (1.8%) serological tests (108 positives). The frequency of serological tests performed decreased during the study period with 329 tests in 2007 and 162 in 2016. Using a serological test, 40 patients have been tested twice, three patients three times and one patient four times. In 10 cases the second serological test was done three to six weeks after the initial antibody test.

The Serological tests performed were either the RIDA®FLUOR Legionella IgG (r-biopharm, 84.8%), in-house methods (4.7%), IFA (Meridian Bioscience Inc., 4.6%), Legionella IFA (Focus Diagnostics, 2.7%), or unknown (3.21%).

Although serological test have their value for epidemiological studies, they are not suitable for clinical settings and acute diagnostics, due to their long turnover for a positive result [25,47]. Moreover a single high titer is only classified as a probable case by the Centers for Disease Control and Prevention (CDC) and the European Centre for Disease Prevention and Control (ECDC) (and accordingly the Swiss Federal Office of Public Health [FOPH]), and single acute phase antibody titers of 1:≥256 cannot distinguish between cases and non-cases [50]. Only a fourfold increase in titer between two tests with 3 to 12 weeks in-between is considered as a confirmed case by the FOPH. However, our data shows that only 45 patients (1.8% of all serological tests) have been tested twice and of those only 10 in the appropriate time period. Hence, a conclusive decision whether a test result was negative (0) or positive (1) could not be made, which would have hampered most of our analyses.

Lastly, since 2018, the titer does not need to be provided on the notification report. Thus, results from serological tests are difficult to interpret and serology is not promoted anymore amongst laboratories for diagnosis of acute LD cases. Therefore, we have decided to exclude these tests from the analysis.

REFERENCES

25. Fields BS, Benson RF, Besser RE. Legionella and Legionnaires' disease: 25 years of investigation. Clin Microbiol Rev. 2002;15(3):506-26.

47. Harrison TG, Taylor AG. Timing of seroconversion in Legionnaires' disease. Lancet. 1988;2(8614):795. doi:10.1016/S0140-6736(88)92442-7.

50. Plouffe JF, File TM, Breiman RF, Hackman BA, Salstrom SJ, Marston BJ et al. Reevaluation of the definition of Legionnaires' disease: use of the urinary antigen assay. Community based pneumonia incidence study group. Clin Infect Dis. 1995;20(5):1286-91.
